# Causal inference techniques for trials of complex interventions

**DOI:** 10.1186/1745-6215-14-S1-P6

**Published:** 2013-11-29

**Authors:** Sabine Landau

**Affiliations:** 1 King's College London, Institute of Psychiatry, London, UK

## 

A statistical framework based on potential outcomes has been developed in which causal effects can be unambiguously defined, and the assumptions needed for their estimation clearly stated. This has also led to the development of new statistical methods that are especially designed for making causal inferences from non-randomised exposures under transparent, less restrictive and more plausible assumptions. I will provide an overview of common research questions in trials of complex interventions (psychological therapies) that target causal effects other than the effects of random treatment offers and are not easily assessed using standard methods. A pertinent example where the effects of non-randomised exposures are of interest is the investigation of treatment effect modification by post-treatment characteristics of therapy such as therapeutic alliance. A second example relates to mechanism investigations in trials of complex interventions. The active components of a complex intervention typically target intermediate process variables in order to improve clinical outcome. Thus it is of interest to investigate empirically whether treatment effect mediation does indeed take place. I will show that such research questions require careful statistical design and analysis to enable consistent estimation of causal parameters of interest. In particular, statistical methods not typically used in routine analysis of clinical trials outcomes, such as instrumental variables methods, can be helpful in addressing further research questions posed by complex interventions. I intend to make the case that causal inference techniques have a lot to offer trials research in complex interventions.

